# Surveillance of transmitted HIV-1 antiretroviral drug resistance in the context of decentralized HIV care in Senegal and the Ebola outbreak in Guinea

**DOI:** 10.1186/s13104-018-3804-9

**Published:** 2018-10-12

**Authors:** Aristid Ekollo Mbange, Djiba Kaba, Abou Abdallah Malick Diouara, Halimatou Diop-Ndiaye, Ndeye Fatou Ngom-Ngueye, Ahmed Dieng, Seynabou Lo, Kine Ndiaye Toure, Mamadou Fall, Wilfred Fon Mbacham, Mariama Sadjo Diallo, Mohamed Cisse, Souleymane Mboup, Coumba Toure Kane

**Affiliations:** 1 Institut de Recherche en Santé, de Surveillance Epidémiologique et de Formation (IRESSEF), Diamniadio, Sénégal; 2 The Biotechnology center, Department of Biochemistry, University of Yaoundé I, Yaoundé, Cameroon; 3 Laboratoire de Bactériologie-Virologie, Centre Hospitalier Universitaire, Aristide Le Dantec/Université Cheikh Anta Diop de Dakar, Dakar, Sénégal; 4 Laboratoire de Biologie Moléculaire Nestor Bangoura/Hélène Labrousse, Hôpital National Donka, Conakry, Guinée; 5 Service de Dermatologie-Vénéréologie Hôpital National Donka/UGAN, Conakry, Guinée; 6 Département de Génie chimique et de Biologie Appliquée, Ecole Supérieure Polytechnique/Université Cheikh Anta Diop de Dakar, Dakar, Sénégal; 7 Laboratoire de Bio-informatique, Université du Québec à Montréal, Montréal, Canada; 8 Centre de Traitement Ambulatoire, Fann, Centre Hospitalier Universitaire, Dakar, Sénégal; 9 Hôpital régional de Saint-Louis, Saint-Louis, Sénégal; 10 Laboratoire de Bactériologie-Virologie CHNU Dalal Jam, Dakar, Sénégal

**Keywords:** Decentralization, Ebola, HIV-1 transmitted drug resistance, First-line regimen, Surveillance, West-Africa

## Abstract

**Objectives:**

Disruption in HIV care provision may enhance the development and spread of drug resistance due to inadequate antiretroviral therapy. This study thus determined the prevalence of HIV-1 transmitted drug resistance (TDR) in settings of decentralized therapy and care in Senegal and, the Ebola outbreak in Guinea. Antiretroviral-naïve patients were enrolled following a modified WHO TDR Threshold Survey method, implemented in Senegal (January–March 2015) and Guinea (August–September 2015). Plasma and dried blood spots specimens, respectively from Senegalese (n = 69) and Guinean (n = 50) patients, were collected for direct sequencing of HIV-1 *pol* genes. The Stanford Calibrated Population Resistance program v6.0 was used for Surveillance Drug Resistance Mutations (SDRMs).

**Results:**

Genotyping was successful from 54/69 (78.2%) and 31/50 (62.0%) isolates. In Senegal, TDR prevalence was 0% (mean duration since HIV diagnosis 4.08 ± 3.53 years). In Guinea, two patients exhibited SDRMs M184V (NRTI), T215F (TAM) and, G190A (NNRTI), respectively. TDR prevalence at this second site, however, could not be ascertained because of low sample size. Phylogenetic inference confirmed CRF02_AG predominance in Senegal (62.96%) and Guinea (77.42%). TDR prevalence in Senegal remains extremely low suggesting improved control measures. Continuous surveillance in both settings is mandatory and, should be done closest to diagnosis/transmission time and with larger sample size.

## Introduction

Genetic imprints of HIV-1 transmitted drug resistance (TDR) in therapy-naïve individuals may readily jeopardize the clinical benefits associated with antiretroviral therapy (ART). Currently this therapy is initiated in developing countries (DC) as a triple combination of two nucleosides reverse transcriptase inhibitors (NRTIs) and a non-NRTI (NNRTI) [1]. These potent drugs primarily aim at suppressing viral load, by interfering with HIV-1 reverse transcriptase (RT) processivity [2]. TDR notably has the potential to increase the risk of virological failures (VF) [3], which detection is crucial to assess response to therapy.

Senegal and Guinea are two neighboring countries of West Africa which together with Central Africa (WCA) globally represents the second most affected region by HIV [4]. In 2016 an estimated 6.1 million people were living with HIV in WCA, although ART coverage remains one of the lowest (33%). Moreover, the handful of patients on therapy are poorly retained on care, which may imply inadequate service delivery with possible consequences on the spread of drug resistance. Especially in fragile health systems like that of Guinea. This country has sustained the overwhelming effects of the Ebola outbreak between 2014 and 2015, with significant repercussions on the provision of HIV services [5]. Another major challenge in most WCA countries is the ineffective decentralization of HIV care. In Senegal, as of 2008, decentralized regions, other than the capital city Dakar, began witnessing upward trends in the proportion (70% in 2013) of patients starting therapy [6]. These settings have been consistently reported with higher rates of VF (23.8–26.0%) associated with acquired drug resistance (ADR) (15.9–17.7%) between 2008 and 2011 [7, 8].

TDR published estimates in Guinea date back to 2009 (8.6%) [9] and in Senegal to 2010 (1.04% protease inhibitors, 4.16% NRTIs) [10]. This study determined and provided updates of TDR prevalence, with the overall goal of improving ART start and monitoring uptake in those challenged settings of HIV care. As such, the presumably long-term efficacy of first-line ART could be safeguarded.

## Main text

### Methods

#### Patients’ enrolment and settings

The WHO threshold survey (TS) method [11] was slightly modified to enroll ART-naïve patients (women and men) irrespective of age (under or over 25 years) and, the number of pregnancies. In Guinea, enrolment was conducted between August and September 2015 at the ambulatory treatment center (ATC) of Donka national hospital (DNH), Conakry. This hospital hosts the largest HIV facility in Guinea and served as one of the Ebola virus treatment centers during the outbreak. In Senegal, patients were enrolled between January and March 2015 at the ATC of Dakar (University Teaching Hospital/UTH of Fann), which currently operates as the reference center for HIV management in Senegal. Recruitment also took place at the regional hospital of Saint-Louis (RHS); Saint-Louis being one of the thirteen decentralized regions for HIV treatment and care in Senegal. Enrolment at respective sites was undertaken at the time of the CD4 threshold strategy of 500 cells/mm^3^ for ART eligibility.

HIV guidelines in Senegal [6, 12, 13] and Guinea [14, 15] are similar in many respects. Individuals willing to take an HIV test undergo a pre-/post-test counselling prior to medical and psychosocial support. Upon entering to care CD4 T-cells are enumerated and three-/six-monthly thereafter (or when clinically indicated). First-line ART is started when CD4 < 500 cells/mm^3^. At the RHS, a social assistant administers adherence counselling, which is mostly ensured by peers or support groups at respective ATCs that track care/treatment defaulters.

At the time of the study, recommended first-line therapies comprised two NRTIs (specifically Zidovudine/Tenofovir + Lamuvidine/Emtricitabine) and one NNRTI (Nevirapine/Efavirenz). Confirmed cases of first-line failures (viral RNA ≥ 1000 copies/ml) are switched to second-line therapies with two NRTIs and one protease inhibitor. For prevention-for-mother-to-child-transmission, both countries adopted option B+.

On-site viral load tests are available/operational since 2013 (ATC/Dakar), 2014 (ATC/DNH), 2016–2018 (RHS) and, provided six-monthly. For ATC/Dakar, these tests were previously offered at the reference Laboratory of Bacteriology–Virology (LBV) of the Aristide Le Dantec UTH in Dakar. Unlike Dakar, resistance testing is lacking in Guinea and decentralized settings of Senegal.

#### Samples processing

All patients in Senegal provided blood specimens (5 ml), collected in EDTA-tubes and centrifuged to harvest plasma that was stored at − 80 °C. In Guinea, blood was spotted (50 µl/spot) onto filter papers (Whatman^®^; 903) before shipping to the LBV where all samples were genotyped. Dried blood spots (DBS) were also kept at − 80 °C until molecular analysis and, their preparation and shipping conditions have been described earlier [8].

#### HIV-1 resistance genotyping

Viral RNA was extracted from plasma and DBS using the QIAmp^®^ Viral RNA Mini Kit (250) (Qiagen, Courtaboeuf France) and NucliSENS easyMag (bioMerieux, Craponne, France), respectively. cDNA Synthesis and PCR (nested-) were carried out in a one-tube reaction containing AMV-reverse transcriptase (Promega, USA). HIV-1 *pol* gene encoding the protease (PR) and partial RT drug-targets was sequenced following the ANRS protocol [16]. Using the Calibrated Population Resistance program v6.0 (http://cpr.stanford.edu), WHO Surveillance Drug Resistance Mutations (SDRMs) [17] were screened uniquely against the first-line antiretroviral-target RT. Contigs were assembled on SeqMan™ II v5.08 (DNASTAR*, Lasergene Konstanz Germany). Consensus sequences were quality-controlled for possible contamination by computing pairwise genetic distances on MEGA v6.06.

#### Phylogenetic and recombination analysis

Multiple sequence alignment was executed with Mafft v7.31 [18] and, included reference sequences from HIV-1 subtypes A-K and several circulating recombinant forms (CRFs) [19]. Phylogenetic trees were inferred by the Maximum Likelihood method with PhyML v3.1 [20], under the GTR + Γ_5_ + I nucleotide substitution model. The Subtree-Pruning-Regrafting heuristic search was applied for optimal tree topology. Branch statistic was computed by the Shimodaira-Hasegawa-approximate likelihood ratio test (SH-aLRT) [21]. Patterns of recombination, notably for outliers, divergent and, long-branch sequences, were determined through Boot-scanning using SimPlot v3.5.1. All trees were read and edited with MEGA.

#### Statistical analysis

Patients’ data were summarized as frequencies, means (± SD) and median (inter-quartiles, IQR) using Epi-info™ v.7.2.1.

### Results

#### HIV-1 transmitted drug resistance in Senegal

**Fig. 1:**
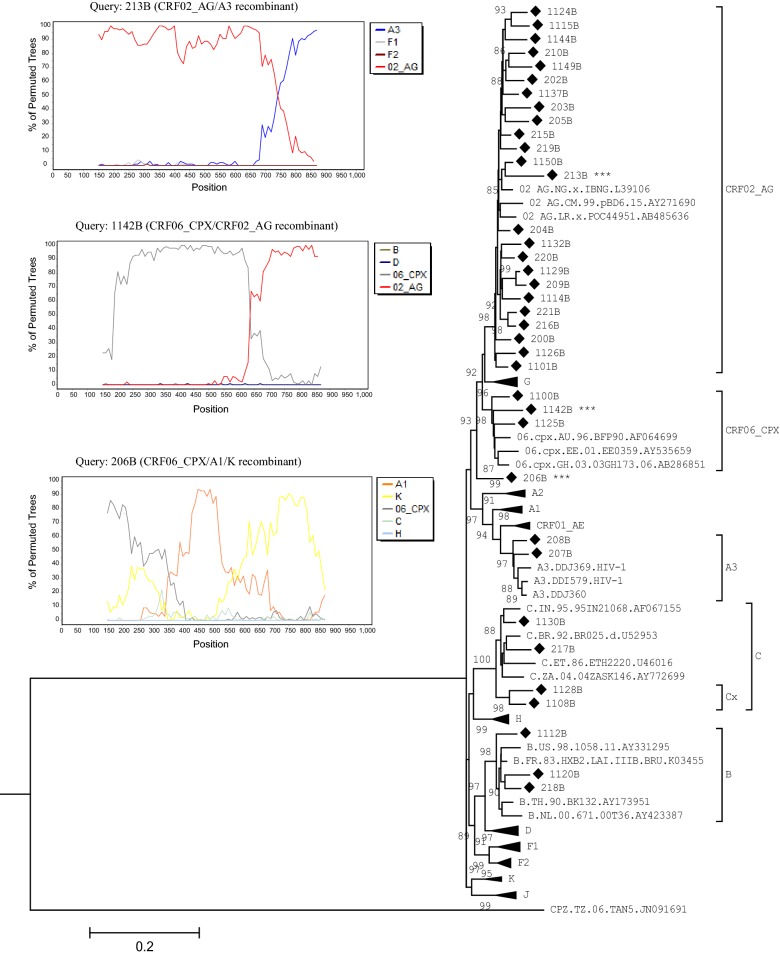
Phylogenetic inference of HIV-1 protease and partial reverse transcriptase sequences from antiretroviral-naïve patients in Senegal. Maximum likelihood analysis, implemented with the PhyML standalone package v3.1, involved 89 nucleotide sequences covering 1026 positions in the final dataset. Branch lengths are measured in the number of substitutions per site. The percentage of trees (SH-aLRT) in which the associated taxa clustered together is shown and values ≥ 85 were significant. The “Cx” clade indicates a possible transmission network between two men (self-reported homosexual and heterosexual). Some clades were collapsed for clarity. Diamond triangles are study field isolates (right panel), of which those with three stars*** are unique recombinant forms (URFs). The left panel depicts boot-scanning plots for each URF queried against representative HIV-1 reference sequences obtained from the Los Alamos HIV database (https://www.hiv.lanl.gov). These sequences are color coded and included A3, CRF02_AG, F1, F2, B, D, CRF06_CPX, A1, K, C, H. Genomic splits or breakpoints were confirmed by reconstruction of phylogenetic trees focused on those unbroken regions (not shown). Boot-scanning was generated in SimPlot v3.5.1 under the Neighbor-Joining algorithm, modelled with the Kimura two-parameter and 100 bootstrap replicates (percentage of permuted trees on the y-axis). Boot-scanning was run with parameters of 50% consensus sequences, 300 base-pair window size, 10 base-pair step size (nucleotides position on the x-axis), and a nucleotide transition/transversion ratio of 2.0

**Fig. 2:**
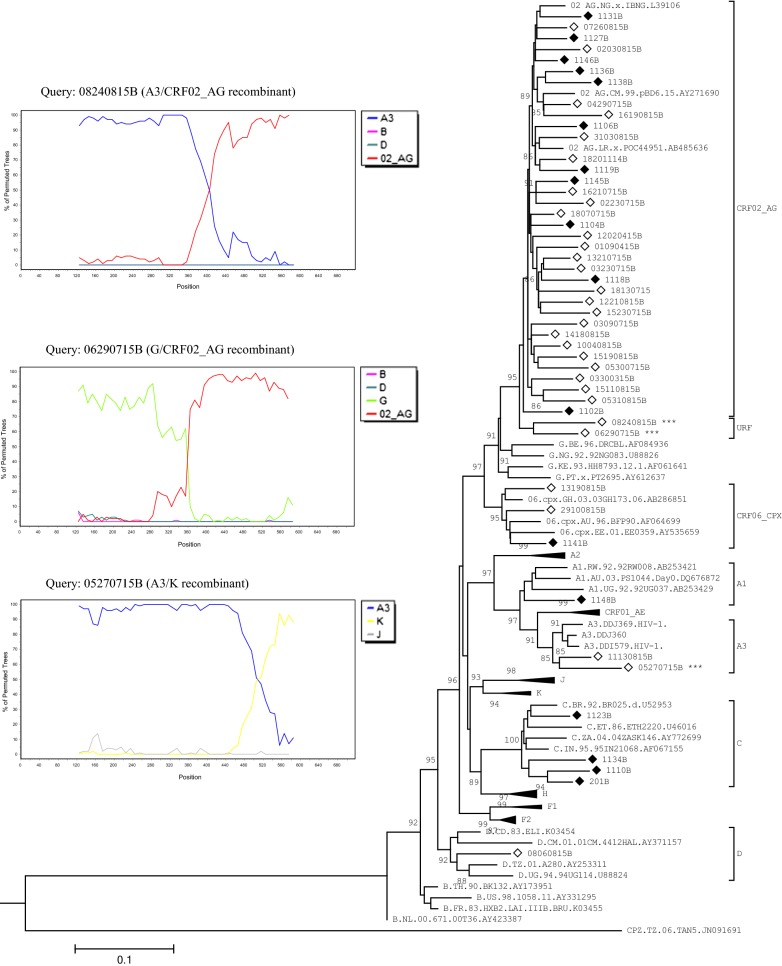
Phylogenetic inference of HIV-1 partial reverse transcriptase sequences from antiretroviral-naïve patients in Senegal and Guinea. Maximum likelihood analysis, implemented with the PhyML standalone package v3.1, involved 98 nucleotide sequences covering 717 positions in the final dataset. Branch lengths are measured in the number of substitutions per site. The percentage of trees (SH-aLRT) in which the associated taxa clustered together is shown and values ≥ 85 were significant. Filled and empty diamond triangles are study field isolates, respectively from Senegal and Guinea. Some clades were collapsed for clarity. Unique recombinant forms (URFs) are indicated in the tree (right panel) with three stars***. The left panel shows Boot-scanning plots for each URF queried against representative HIV-1 reference sequences obtained from the Los Alamos HIV database (https://www.hiv.lanl.gov). These sequences are color coded and included A3, CRF02_AG, B, D, G, K, J. Genomic splits or breakpoints were confirmed by reconstruction of phylogenetic trees focused on those unbroken regions (not shown). Boot-scanning was run with the settings described in Fig. 1 (legends); at the difference that the window size was set at 250 base-pair

In total 69 ART-naïve patients were enrolled at the ATC/Dakar (68.11%) and RHS (31.88%). The median age of the study population was 37 years (IQR 32–45) with females being mostly represented (65.2%, 45/69) (Table 1). At inclusion, mean time since HIV diagnosis (MTSD) was 4.08 ± 3.53 years. Fifty-four of 69 (78.26%) isolates were successfully genotyped in their PR-RT (53.62%, 37/69) and RT (24.63%, 17/69) genomic regions. No SDRM was found leading to an estimated prevalence of 0% TDR. The CRF02_AG variant was the most widespread (62.96%, 34/54) followed by, subtype C (14.81%, 8/54), B and CRF06_CPX (5.55% each, 3/54), A3 (3.70%, 2/54) and, A1 (1.85%, 1/54) (Figs. 1 and 2). The designated “Cx” cluster branched basal to the main C clade with strong support (SH-aLRT = 98) suggesting a transmission network. Three unique recombinant forms (URFs) were identified (5.55%): CRF02_AG/A3, CRF06_CPX/CRF02_AG, CRF06_CPX/A1/K (Fig. 1).

**Table 1:** Demographic and clinical characteristics of antiretroviral-naïve patients in Senegal and Guinea

Variables/Categories	Frequency (%)/Median with interquartile [IQR] or Mean with Standard Deviation (SD)	
	Senegal (Dakar + Saint-Louis) N = 69	Guinea (Donka) N = 50
Gender		
Female	45 (65.2)	29 (58.0)
Male	24 (34.8)	21 (42.0)
Age (years), median	37.0 [32.0–45.0]	34.0 [26.0–43.0]
Age groups		
< 25	6 (8.7)	11 (22.0)
25–39	35 (50.7)	24 (48.0)
≥ 40	28 (40.6)	15 (30.0)
Mean years since HIV diagnosis	4.08 ± 3.53	0.74 ± 1.39
Years since HIV diagnosis		
< 1	19 (27.54)	47 (94.0)
1–3	13 (18.84)	–
> 3	34 (49.28)	–
Missing	3 (4.35)	3 (6.0)
CD4 (cells/mm^3^), Median recent three months	537.0 [461.0–643.0]	307.5 [185.0–434.0]
CD4 groups		
≤ 500	22 (31.88)	31 (62.0)
> 500	39 (56.52)	5 (10.0)
Missing	8 (11.59)	14 (28.0)
Viral load* (log_10_ copies/ml), median	–	5.52 [4.84–6.31] (range 2.40–12.80)
Viral load groups		
< 3.30	–	4 (8.0)
≥ 3.30 < 4.0	–	2 (4.0)
≥ 4.0	–	44 (88.0)

*Quantitation of plasma viral RNA particles was done using the Generic HIV Viral Load Kit (Biocentric^®^)

#### HIV-1 transmitted drug resistance in Guinea

At DNH, included patients totaled 50. The median age was 34 years (IQR 26–43) and females constituted more than half of the study population (58.0%, 29/50) (Table 1). Patients in their quasi-totality (94.0%, 47/50) were diagnosed with HIV less than a year before sampling. All Guinean HIV-1 RT sequences were genotyped with a 62.0% (31/50) success rate. One isolate harbored the NNRTI-G190A SDRM, whereas another exhibited dual-class resistance carrying NRTI-M184V, T215F (thymidine-analogs mutation) and, NNRTI-K103N. CRF02_AG constituted 77.42% (24/31) of all HIV-1 infections followed by, CRF06_CPX (6.45%, 2/31), subtype D and sub-subtype A3 (3.22% each, 1/31) (Fig. 2). Equally three mosaics forms were characterized (9.67%): A3/CRF02_AG and G/CRF02_AG forming a weak (SH-aLRT < 85) monophyletic cluster and, A3/K (Fig. 2).

### Discussion

The 0% TDR prevalence found in Senegal comforts previous studies from this site that reported no NNRTIs mutations and, a decline in NRTIs TDR (4.16–0%) between 1998 and 2006/2007 [10, 22]. This extremely low rate is contrary to that observed in a meta-analysis showing a 3.5% rate at 5–7 years after ART rollout in WCA [23] and, in Liberia (Monrovia) in 2013 (5.9%) [24]. Such stable prevalence most likely reflects substantial inputs from AIDS control programs to minimize HIV risky behaviors. Furthermore, improvement in treatment compliance over the years and viral load control, at least at the ATC/Dakar, may have contributed to this result.

Compared to the ATC/Dakar, viral load and resistance testing at the time of this investigation were hardly performed at the RHS. In a concurrent cross-sectional study conducted in these two facilities, we showed significant odds of VF and ADR to first-line ART, taken for ≥ 12 months, at the RHS (unpublished data). Despite the consistent rollout of antiretrovirals at decentralized sites since 2008, the 27.53% therapy-naïve cases at the RHS harbored no SDRM. This observation underscores the importance of preventing new infections in a context of inadequate therapy monitoring. Nonetheless, the magnitude of TDR at the RHS remains to be ascertained because of the few isolates screened. The restrictive criteria of WHO-TS to apply in antenatal settings of low HIV prevalence may explain this limitation [25]. At Saint-Louis, this prevalence accounted for 0.3% among pregnant women [13].

The underrepresented proportion of patients < 25 years old, especially in Senegal, might be perceived as an underestimation of TDR prevalence. This criterion and, primigravidity have shown limited sensitivities in identifying recent infections [25, 26]. The current study was implemented before the test-and-treat policy. In Malawi, an excellent study revealed higher misclassifications rates for antiretroviral start with the CD4 threshold of 500 cells/mm^3^; namely between 350 and 650 cells/mm^3^ with single measurements [27]. As such, the reliability of the CD4 count as a marker of recent infection (CD4 > 500 cells/mm^3^) may have implications for the WHO-TS in DC. Furthermore, this approach may defer treatment start, thereby increasing the likelihood of onward TDR [28]. Scrutinizing ART starters for pre-treatment drug resistance [29] therefore remains central for the optimization of first-line therapies in DC. The inconsistency in median CD4 (537 cells/mm^3^) and MTSD (4.08 ± 3.53 years) in Senegalese patients may signify longer infections. This finding contradicts that seen in Guinean patients (median CD4, 307.5 cells/mm^3^; MTSD, 0.74 ± 1.39 years). In Tanzania, older patients (38.9 ± 10.1 years) presented a 19% TDR prevalence (mean CD4, 478 ± 223 cells/mm^3^, MTSD, 1.45 ± 0.23 years) [26]. Hence, sampling of SDRMs should be done closest to diagnosis/transmission time. Indeed, prolonged infections in the absence of drug pressure tend to recede to levels undetectable by Sanger sequencing [30].

The WHO-TS strategy requires a minimum of 34–47 samples to report TDR in DC. Regardless of this criterion and, in spite of the Ebola crisis it could be argued that TDR rate in Guinea has not known any increase from 2009 when the last survey was conducted [9]. The prevalence seems to have dropped from 8.6% to now 6.45%, which is below that seen in Niger (8.3%) [31]. Further studies are needed, however, to support this finding as TDR prevalence in Guinea could not be scored as low (< 5%), moderate (5–15%) or, high (> 15%) [11]. Only few specimens could be genotyped, possibly because of low viral loads or sub-optimal storage conditions leading to viral genome breakages. Notably as DBS shipment to Senegal was delayed owing to fear of the Ebola virus.

SDRMs detected in Guinea may cause high-level resistance at initiation to Zidovudine (T215F), Lamivudine (M184V) and, Nevirapine/Efavirenz (G190A/K103N). These resistant genotypes have been previously described in therapy-naïve patients at DNH [9] and, with T215 revertants may persistently survive in drug-free environments unlike M184V and T215F [32, 33]. Ibe et al. [34] proposed such fitness property could be attributable to mutations (compensatory) occurring in other genomic regions.

Molecular phylogenetic confirmed CRF02_AG predominance in Western-Africa [35]. Recombination profiling warrants detailed near/full-length genome sequencing to assess the extent of HIV-1 diversity in our study settings [36].

### Conclusion

The prevalence of HIV-1 TDR in Senegal was extremely low but arguably moderate in Guinea, despite major challenges in the provision of HIV-related services. Further surveillance studies are needed especially in decentralized areas where treatment monitoring uptake is inadequate. Surveillance of TDR likewise should be enhanced in Guinea as disruption of health care due to the Ebola epidemic may have led to increased therapeutic failures.

## Limitations

The sample size of this survey was not sufficiently informative of HIV-1 TDR dynamic at the decentralized and Ebola sites. Secondly, infections tended to be chronic in our sampling setting of Senegal, thus cautioning against extrapolation of the current 0% TDR. The use of conventional sequencing limits the detectability of resistant minority variants, which may have underestimated TDR prevalence.

## Abbreviations

ADR: acquired drug resistance
aLRT: approximate likelihood ratio test
ART: antiretroviral therapy
CD4: cluster of differentiation 4
CRF: circulating recombinant forms
DBS: dried blood spots
GTR: general time reversible
MAFFT: multiple alignment fast fourrier transformation
SDRM: surveillance drug resistance mutations
TDR: transmitted drug resistance
TS: threshold survey
URF: unique recombinant forms
VF: virological failure
WHO: world health organization
